# Hepatocyte RXRalpha deficiency in matured and aged mice: impact on the expression of cancer-related hepatic genes in a gender-specific manner

**DOI:** 10.1186/1471-2164-9-403

**Published:** 2008-08-28

**Authors:** Minglei Guo, Lei Gong, Lin He, Lois Lehman-McKeeman, Yu-Jui Yvonne Wan

**Affiliations:** 1 Department of Pharmacology, Toxicology & Therapeutics, University of Kansas Medical Center, Kansas City, KS, 66103, USA; 2 Discovery Toxicology, Bristol-Myers Squibb Company, Princeton, NJ 08543, USA

## Abstract

**Background:**

The occurrence of liver cancer is higher in males than in females, and the incidence increases during aging. Signaling pathways regulated by retinoid × receptor α (RXRα) are involved in hepatocellular carcinogenesis. The phenotype of hepatocyte RXRα deficient mice is different between genders. To explore the impact of hepatocyte RXRα deficiency on gender-dependent hepatic gene expression, we compared the expression profiles of cancer-related genes in 6 and 24 month old male and female mice.

**Results:**

In 6 month old mice, male mutant mice showed more cancer-related genes with alteration in mRNA levels than females did (195 vs. 60). In aged mice (24 month), female mutant mice showed greater deviation in mRNA expression levels of cancer-related genes than their male counterparts (149 vs. 82). The genes were classified into five categories according to their role in carcinogenesis: apoptosis, metastasis, cell growth, stress, and immune respnse. In each category, dependent upon age and gender, the genes as well as the number of genes with altered mRNA levels due to RXRα deficiency varies.

**Conclusion:**

The change in hepatic cancer-related gene expression profiles due to RXRα deficiency was gender- and age-dependent. The alteration of mRNA levels of cancer-related genes implied that aberrant RXRα signaling could potentially increase the risk of liver cancer and that retinoid signaling might contribute to gender- and age-associated liver cancer incidence.

## Background

RXRs (Retinoid × Receptors), belonging to the nuclear receptor superfamily, play important roles in detoxification, apoptosis, differentiation, and proliferation through hetero-dimerizing with other nuclear receptors [1]. RXR α, β, and γ are the receptors for retinoids, and have been used to prevent and treat cancer. RXRα is the most prevalent receptor expressed in liver. Aberrant RXRα-induced pathways have been implicated as possible mechanisms for the development of hepatocellular carcinoma [2,3]. Hepatocyte-specific RXRα-deficient mice were first generated by Wan etc. [4,5]. Although hepatocyte RXRα deficiency does not show an obvious phenotype, many metabolic pathways including fatty acid, cholesterol, and xenobiotic are compromised due to hepatocyte RXRα deficiency. Furthermore, shortened hepatocyte lifespan and impaired capacity for liver regeneration after partial hepatectomy are detected in hepatocytes that do not express RXRα [6]. These findings indicate that hepatocyte RXRα is not only important for liver metabolism, but also in control of hepatocyte proliferation and survival.

The impact of RXRα deficiency on the expression of RXRα target genes is gender dependent. The expression of cytochrome P450 (CYP450) genes including Cyp4a, 3a, and 2b are differentially expressed in male and female hepatocyte RXRα-deficient mice [7]. Using sex hormone treatments, we have previously shown that male hormones might have an impact on regulating RXRα-mediated signaling [7]. In addition to gender, aging also imposes significant changes on nuclear receptor-mediated gene expression in hepatocytes [8]. Nuclear receptor signaling pathways are in hypo-functioning status in an aged person's peripheral blood mononuclear cells [9]. The incidence of liver cancer is much higher in males than in females, and increases with aging. Based on these observations, we hypothesize that aberrant hepatocyte RXRα signaling might have complex repercussions on cell biological activities and contribute to the risk of liver carcinogenesis in an age and gender dependent manner.

To study the impact of hepatoctye RXRα deficiency on cancer-related gene expression in each gender, we have performed microarray analyses using livers derived from 6 and 24 month old male and female wild type and hepatocyte RXRα-deficient mice. It is generally recognized that 6 month old mice are mature and 24 month old mice are aged [10,11]. We used Ingenuity Pathway software to identify cancer-related genes. Generally, these genes can be classified into five categories that are associated with carcinogenesis: 1) apoptosis; 2) stress response; 3) cell migration; 4) cell cycle/growth regulation; and 5) immune response. Our data demonstrated that in 6 month old mice, hepatocyte RXRα deficiency resulted in more changes (both in number and fold) of gene expression profiles in male than in female mice; in contrast, in aged mice (24 month old), the pattern was reversed with females showing more changes in genetic expression profiles than their male counterparts. Our data provide a database for identification of candidate genes that might account for gender-, age-, and retinoid signaling-associated liver cancer development.

## Results and discussion

In 6 month old hepatocyte RXRα deficient mice, 195 genes found in male mice livers had altered expression patterns while 60 genes had changed expression patterns in female mouse livers. In contrast to the matured mice, in aged mice the number of genes that had altered expression patterns due to hepatocyte RXRα deficiency was higher in female (149) than in male (82) mouse livers (table 1). Our data suggest that hepatocyte RXRα deficiency has a greater impact on males than females in young mice. When mice are aged, the impact is greater in female than in male mice.

**Table 1:** Numbers of cancer related genes with altered mRNA levels.

Comparison	6 month old mice (KO vs. WT)		24 month old mice (KO vs. WT)	
Gender	male	female	male	female

apoptosis	11	6	10	5
metastasis	22	6	9	8
stress inducible	8	2	4	2
cell growth	39	11	9	22
immune response	15	4	10	24
unclassified	100	29	42	61
total	195	60	82	149

To validate the microarray results, we selected 10 genes to quantify mRNA levels by qRT-PCR. All qRT-PCR analyses were performed on the same samples used for the microarray experiments. Table 2 summarized the fold change in mRNA levels detected by qRT-PCR and microarray experiments. Both methods detected similar trends of expression (up or down regulation) although the fold changes may not be the same. There were only a few exceptions i.e. FKBP5 and USP2. The difference in fold change may reflect the sensitivity difference between the two methods. Generally, the results from both methods were consistent.

**Table 2:** Validation the changes of mRNA levels in selected genes.

Comparison	6 month old mice KO vs. WT		24 month old mice KO vs. WT		Methods
Gender	male	female	male	female	

FKBP5	4.26	2.63	2.81	(-)	micro-array
	3.26	2.23	(-)	(-)	qRT-PCR
USP2	3.46	2.17	3.97	(-)	micro-array
	3.14	2.2	(-)	(-)	qRT-PCR
Caspase 8	-2.04	(-)	-2.31	(-)	micro-array
	-1.98	(-)	-3.08	(-)	qRT-PCR
THBS1	-7.11	(-)	(-)	(-)	micro-array
	-22.21	(-)	(-)	(-)	qRT-PCR
SERPINE1	-23.48	(-)	(-)	(-)	micro-array
	-11.76	(-)	(-)	(-)	qRT-PCR
FOS	-5.74	(-)	(-)	(-)	micro-array
	-9.94	(-)	(-)	(-)	qRT-PCR
ATF3	-33.85	(-)	(-)	(-)	micro-array
	-24.32	(-)	(-)	(-)	qRT-PCR
DNAJB1	-11.30	(-)	(-)	(-)	micro-array
	-6.10	(-)	(-)	(-)	qRT-PCR
EGR1	-15.88	(-)	(-)	(-)	micro-array
	-9.33	(-)	(-)	(-)	qRT-PCR
BTG2	-5.26	(-)	(-)	(-)	micro-array
	-9.34	(-)	(-)	(-)	qRT-PCR

(-): No significant change on mRNA expression level.

Many biological processes can be compromised during carcinogenesis. These processes include resistance to apoptosis, unlimited replication potential, self-sufficient growth signal, insensitivity to negative regulators, sustained angiogenesis, and impaired tissue remodeling, all which influence cancer cells to metastasize [12]. In addition, cell-host interactions such as immune response and stress response pathways have been demonstrated to play important roles in carcinogenesis [13,14]. To reflect these processes, we classified the cancer-related genes, which changed their expression level due to hepatocyte RXRα deficiency, into the five categories: apoptosis, migration, cell growth regulation, stress induction, and immune response.

### Apoptosis-related genes

In the group of apoptosis-related genes, two general trends were noted in table 3. (1) The number of genes with varied mRNA levels was always higher in male mutant mice than in female mutant mice. In 6 month old mice, 11 apoptosis-associated genes had altered mRNA levels in male mutant mice while in females; only 6 apoptosis genes had changed expression levels. In 24 month old mice, 10 apoptosis-associated genes had significant changes in mRNA levels in male mutant mice; while in females, the number decreased to 5. (2) Most anti-apoptosis genes had increased mRNA levels; on the contrary, most pro-apoptosis genes had decreased expression levels in due to RXRα deficiency. In male mice, all 4 anti-apoptosis genes in 6 month old mice and 4 out of 5 anti-apoptosis genes in 24 month old mice had increased mRNA levels. On the other hand, 4 out of 7 and 1 out of 1 pro-apoptosis genes decreased in mRNA expression level, respectively, in 6 and 24 month old male mice. The same trend was also found in female mutant mice. A combination effect of up-regulated anti-apoptosis genes and down-regulated pro-apoptosis genes indicated that RXRα-deficient hepatocytes have a more resistant capacity to apoptosis and that hepatocyte RXRα deficiency might have a pro-survival effect.

**Table 3:** Fold changes of the mRNA levels of the apoptosis-related genes in male and female hepatocyte RXRα-deficient mouse livers.

6 month male mice (KO *vs*. WT)			6 month female mice (KO *vs*. WT)		
Name	Fold Change	function	Name	Fold Change	function

FKBP5	4.26	anti-apoptosis	FKBP5	2.63	anti-apoptosis
USP2	3.46	anti-apoptosis	USP2	2.17	anti-apoptosis
CFLAR	2.56	anti-apoptosis	BCL6	2.14	anti-apoptosis
BGN	2.4	anti-apoptosis	SERPINA3K	2.06	anti-apoptosis
BIK	4.41	pro-apoptosis	BGN	-2.12	anti-apoptosis
ACVR2B	3.55	pro-apoptosis	ANP32A	-2.01	pro-apoptosis
BNIP2	2.09	pro-apoptosis			
CASP8	-2.04	pro-apoptosis			
EMP2	-2.7	pro-apoptosis			
DUSP6	-4.26	pro-apoptosis			
BCL2L11	-5.61	pro-apoptosis			
					
24 month male mice (KO *vs*. WT)			24 month female mice (KO *vs*. WT)		

Name	Fold Change	function	Name	Fold Change	Function

USP2	3.97	anti-apoptosis	IER3	2.23	anti-apoptosis
FKBP5	2.81	anti-apoptosis	BCL6	2.08	anti-apoptosis
IER3	2.8	anti-apoptosis	BIK	2.22	pro-apoptosis
DUSP1	2.3	anti-apoptosis	WNK1	-2.35	pro-apoptosis
BIRC4	-3.19	anti-apoptosis	DIABLO	-2.87	pro-apoptosis
CASP1	2.83	pro-apoptosis			
UBE1L	2.36	pro-apoptosis			
ZBTB16	2.19	pro-apoptosis			
SOX9	-2.09	pro-apoptosis			
CASP8	-2.27	pro-apoptosis			

Among the anti-apoptosis genes, FKBP5 (FK506 binding protein 5) mRNA levels were increased by 4.26- and 2.63-fold in 6 month old male and female mice, respectively, due to hepatocyte RXRα-deficiency, and the data were confirmed by real-time PCR. FKBP5 is a co-chaperone molecular which interacts with HSP90 (Heat Shock Protein 90) [15]. Its roles include up-regulating the NF-κB pathway and stimulating Bcl2 transcription. FKBP5 could be up-regulated by androgen, glucocorticoids, and progestin hormones [15]. There is no evidence showing that FKBP5 is directly associated with RXRα-mediated signaling. However, RXRα could negatively modulate androgen signaling through binding androgen receptors directly [16]. It is possible that in RXRα-deficient hepatocytes, the androgen-mediated signaling could have enhanced activation levels compared with wild-type mice thus leading to higher expression levels of FKBP5.

Another anti-apoptosis gene, USP2 (Ubiquitin Specific Peptidase 2), also showed increased mRNA levels by 3.46- and 2.17-fold in 6 month old hepatocyte RXRα deficient male and female mice, respectively. USP2 is a de-ubiquitinase protein and increases Mdm2 (mouse double minute 2) [17] and FAS (fatty acid synthase) protein stability [18]. Since Mdm2 is responsible for p53 degradation, USP2 could negatively regulate the p53 pathway activity through up-regulation of Mdm2. In prostate cancer cells, USP2 interacts with and stabilizes FAS, which is often over-expressed in biologically aggressive human tumors. Functional inactivation of USP2 results in decreased FAS protein and enhanced apoptosis in prostate cancer [18]. As with FKBP5, USP2 is also up-regulated by androgen. The similarly elevated expression patterns for FKBP5 and USP2 genes suggest that they are likely regulated by the same mechanism, possibly up-regulation of androgen signaling activity due to RXRα deficiency. In addition, the qRT-PCR results showed that in both genders, the levels of FKBP5 and USP2 mRNA were not increased in 24 month old mutant mice probably due to decreased androgen level in aged mice.

We previously showed that hepatocyte RXRα-deficient mice have increased serum cholesterol and triglyceride levels [5], indicating an altered fatty acid metabolism pathway. Our results implied elevated serum triglyceride and cholesterol levels might in part be due to increased activity of FAS because of up-regulation of USP2. Collectively, the changed trends in apoptosis related genes implied that RXRα-deficient hepatocytes have an increased resistance to apoptosis.

### Migration-related genes

Genes in this group play important roles in cell migration and angiogenesis and are associated with metastasis, a key feature of malignant cancer cells. Generally, the trends observed in this group were different depending upon age and gender (table 4).

**Table 4:** Fold changes of the mRNA levels of the metastasis-related genes in male and female hepatocyte RXRα-deficient mouse livers.

6 month male mice (KO *vs*. WT)			6 month female mice (KO *vs*. WT)		
Name	Fold Change	Function	Name	Fold Change	function

ARHGDIB	-2.05	anti-metastasis	BRMS1	-2.16	anti-metastasis
KRT18	-2.15	anti-metastasis	CD36	-2.88	anti-metastasis
CD36	-2.16	anti-metastasis	MYO10	2.63	pro-metastasis
KRT19	-2.43	anti-metastasis	ROCK1	-2.24	pro-metastasis
THBS1	-7.11	anti-metastasis	CTTN	-2.88	pro-metastasis
SERPINE1	-23.48	anti-metastasis	ITGA6	-3.64	pro-metastasis
CAV1	2.86	pro-metastasis			
FN1	2.67	pro-metastasis			
ID2	2.65	pro-metastasis			
DDEF1	2.52	pro-metastasis			
CLCA1	2.33	pro-metastasis			
S100A10	-2.09	pro-metastasis			
ADAM12	-2.12	pro-metastasis			
CNN2	-2.16	pro-metastasis			
PLAUR	-2.25	pro-metastasis			
PLAT	-2.30	pro-metastasis			
NEDD9	-2.39	pro-metastasis			
SPP1	-2.75	pro-metastasis			
CTGF	-2.90	pro-metastasis			
VIM	-3.25	pro-metastasis			
LGALS7	-3.79	pro-metastasis			
EPHA2	-4.71	pro-metastasis			
					
24 month male mice (KO *vs*. WT)			24 month female mice (KO *vs*. WT)		

Name	Fold Change	Function	Name	Fold Change	function

TIMP4	4.07	anti-metastasis	DDR1	-2.20	anti-metastasis
ANK3	2.07	anti-metastasis	TFF3	11.22	pro-metastasis
LGALS3	2.00	anti-metastasis	ADAM15	3.38	pro-metastasis
TFF3	4.05	pro-metastasis	MMP9	2.49	pro-metastasis
CTGF	2.22	pro-metastasis	ITGB1	2.48	pro-metastasis
MT1E	2.19	pro-metastasis	CSF2RB	2.32	pro-metastasis
CAV1	2.15	pro-metastasis	FOXM1	2.13	pro-metastasis
PLAUR	2.03	pro-metastasis	MYO6	-4.70	pro-metastasis
PTP4A3	-2.55	pro-metastasis			

In 6 month male RXRα-deficient mice, 22 genes showed significant change in mRNA levels. Among those, 6 out of 6 anti-metastasis genes showed reduction of mRNA levels because of RXRα deficiency. Other genes associated with pro-metastasis showed either up- or down-regulation in mRNA levels due to RXRα deficiency. In female hepatocyte RXRα-deficient mice, only 6 migration-related genes showed significant changes in their mRNA level.

At 24 month of age, male and female mutant mice showed 9 and 8 genes with alteration in their mRNA levels, respectively. Thus, in aged mice, the difference in the number of genes with deviated mRNA levels is no longer obvious between genders; in contrast, at 6 month of age, the numbers of genes with altered mRNA levels in male and female mutant mice were 22 and 6, respectively. Another striking observation was that many pro-metastasis genes increased their mRNA levels in aged RXRα deficient mice. In contrast, very few anti-metastasis associated genes showed change in mRNA levels in aged mice.

In the group of anti-metastasis associated genes, the levels of CD36, THBS1 (thrombospondin 1), and SERPINE1 (Serpin Peptidase Inhibitor) mRNA decreased by 2.16-, 7.11-, and 23.48-fold, respectively, in 6 month old male mutant mice. Real time PCR results showed that THBS1 and SERPINE1 were down-regulated in mRNA levels by 22.21-, and 11.76-fold, respectively. THBS1 is the receptor for CD36 and a potent angiogenesis inhibitor. Down-regulation of THBS1 has been suggested to increase tumor growth and metastasis by modulating angiogenesis in a variety of tumor types [19]. SERPINE1, also named PAI-1 (plasminogen activator inhibitor-1), has been used in gene therapy for inhibition of melanoma metastasis [20]. There were also some pro-metastasis genes, such as CAV1 (caveolin 1) and FN1 (fibronectin 1), which exhibited increased mRNA levels by 2.86- and 2.67-fold, respectively, in 6 month old male mice. On the contrary, in female hepatocyte RXRα-deficient mice, those genes, except CD36, did not show changes in expression levels. These observed expression patterns indicate that RXRα deficiency had a greater impact on metastasis related gene expression in males than in female mice at an earlier stage of life. It also suggests that hepatocytes in male mutant mice might have more cell movement ability than wild type hepatocytes.

Our data indicated that RXRα-deficient hepatocytes might have more metastasis ability than normal hepatocytes. Male mutant mice at 6 month of age had 22 genes with changed their mRNA levels. All 8 anti-metastasis genes showed decreased mRNA levels. When mice were 24 month old, the up-regulation of mRNA levels in pro-metastasis related genes became more robust in hepatocyte RXRα-deficient mice. It has been revealed that RXRα ligands could inhibit cell migration through deregulation of matrix metalloproteinase-9 or TIMP-1 production [21]. Down-regulation of THBS1 and SERPINE1 when retinoid signaling is compromised provide another mechanism by which retinoids might have an anti-metastasis role. Our data also suggest that the impact of RXRα on metastasis is gender and age dependent.

### Stress response-related genes

Several HSP (Heat Shock Protein) genes decreased their mRNA levels due to RXRα deficiency (table 5). At 6 month of age, male and female mutant mice had 6 and 2 HSP genes with decreased mRNA levels, respectively. At 24 month of age, two HSP genes, HSPB1 (heat shock 27 kDa protein 1) and HYOU1 (hypoxia up-regulated 1), showed decreased mRNA levels due to RXRα deficiency.

**Table 5:** Fold changes of the mRNA levels of the stress-inducible genes in male and female hepatocyte RXRα-deficient mouse livers.

6 month male mice (KO *vs*. WT)			6 month female mice (KO *vs*. WT)		
Name	Fold Change	function	Name	Fold Change	function

GADD45G	3.89	stress inducible	HSPA8	-2.50	stress inducible
DDIT4	2.30	stress inducible	HSPB1	-4.66	stress inducible
DNAJB4	-2.39	stress inducible			
HSPA1B	-3.42	stress inducible			
HSPB1	-3.86	stress inducible			
HSPA1A	-10.29	stress inducible			
DNAJB1	-11.30	stress inducible			
ATF3	-33.85	stress inducible			
					
24 month male (KO *vs*. WT)			24 month female (KO *vs*. WT)		

Name	Fold Change	function	Name	Fold Change	function

GADD45G	2.86	stress inducible	HIF1A	2.50	stress inducible
DDIT4	2.18	stress inducible	HIPK2	-2.22	stress inducible
HSPB1	-2.59	stress inducible	HSPB1	-2.62	stress inducible
HYOU1	-2.75	stress inducible	HYOU1	-3.54	stress inducible

The HSP gene family is highly conserved in structure from C. elegans to humans. HSP genes constitute the cellular protection mechanism and can be induced by various physical, chemical, and biological factors. In 6 month old RXRα-deficient male mice, DNAJB1 (Dnaj homologue, subfamily B, member 1), HSPB1, HSPA1A (Heat Shock 70 KDa Protein 1A), and HSPA1B (Heat Shock 70 KDa Protein 1B) had reduced mRNA levels by 11.30-, 10.29-, 3.86-, and 3.42-fold, respectively. This coordinated down-regulation of the HSP family genes indicated that these genes were regulated by common mechanisms. In 6 month old hepatocyte RXRα-deficient female mice, only 2 genes (HSPA8 and HSPB1) had reduced mRNA levels of 2.50- and 4.66-fold, respectively. Thus, hepatocyte RXRα deficiency has a greater impact on HSP gene expression in male than in female mice. In aged mice, there was no gender difference in the expression pattern of the HSP family genes related to RXRα deficiency. HSPB1 and HYOU1 both exhibited decreased mRNA levels in male and female aged mutant mice to a similar extent. HSPB1 mRNA levels were consistently decreased in both male and female mutant mice of both age groups.

Some small HSP genes expression levels such as HSP27 can be up-regulated by RXR/RAR heterodimer in lens [22]; furthermore, RXR ligand 9-cis RA (retinoid acid) increases the HSP gene expression in Jurkat cells [23]. Rocchi, P., et al. suggested that the expression of HSPB1 could be up-regulated by androgen ablation [24]. Another report revealed that exogenous androgen treatment could delay stress response by decreasing the expression of HSP70 [25]. Down-regulation of these HSP genes implied that RXRα-deficient hepatocytes had a reduced protective ability and might be more susceptible to injury resulted from external stimuli compared with wild type hepatocytes. It is possible that there was increased androgen signaling activity due to RXRα deficiency because RXRα is a negative regulator for the androgen pathway, leading to inhibition of HSP family mRNA expression. Another phenomenon that we observed is that the number of HSP family genes which showed alteration in mRNA levels is higher in male than in female when mice are 6 month old. However, this gap decreased when mice were aged. Since androgen levels are higher in male than in female and decrease during aging, the physiological changes of androgen levels may account for this gender- and age-dependent gene expression pattern. It has been shown that RXRα-deficient hepatocytes have a shortened lifespan [6]. Our data implied down-regulation of HSP genes expression in RXRα-deficient hepatocytes might result in decreased cell protection ability and consequently render the cells prone to death.

### Cell growth regulation-related genes

Retinoids could arrest cell cycle progression and induce apoptosis in many types of cancer cells through activation of RXRs. RXRα signaling plays critical roles in cell growth and differentiation. In RXRα hepatocyte-deficient mice, many genes associated with cell growth had changes in their mRNA levels. Again, this difference in gene expression pattern is gender and age dependent (table 6).

**Table 6:** Fold changes of the mRNA levels of the cell growth-related genes in male and female hepatocyte RXRα-deficient mouse livers.

6 month male mice (KO *vs*. WT)			6 month female mice (KO *vs*. WT)		
Name	Fold Change	function	Name	Fold Change	function

LCN2	6.37	oncogene	LCN2	4.57	oncogene
PIM1	4.10	oncogene	GFRA1	2.15	oncogene
MAFB	4.07	oncogene	PTTG1	-4	oncogene
ERBB3	2.05	oncogene	PROM1	-5.61	oncogene
REL	-2.34	oncogene	PLAGL1	-2.09	suppressor
MDM2)	-2.47	oncogene	KLF6	-2.19	suppressor
JUN	-2.87	oncogene	WWOX	-2.2	suppressor
RHOB	-2.93	oncogene	CAPG	-2.23	suppressor
PTTG1	-2.95	oncogene	KLF4	-2.32	suppressor
RHOC	-3.06	oncogene	GJA1	-2.91	suppressor
ROS1	-3.14	oncogene	GSN	-3.18	suppressor
SERTAD1	-3.7	oncogene	PEG3	-5.96	suppressor
PML	-3.9	oncogene	TPM1	-11.95	suppressor
ETS2	-4.97	oncogene			
FOS	-5.74	oncogene			
MYC	-7.7	oncogene			
SOCS3	3.85	suppressor			
MAD1L1	3.73	suppressor			
TCF21	2.82	suppressor			
CEBPD	2.77	suppressor			
SSBP2	2.27	suppressor			
NDRG1	2.19	suppressor			
HMGN1	2.1	suppressor			
SLC22A18	2.08	suppressor			
AIM1	2.05	suppressor			
KSR1	2.04	suppressor			
CDKN1A	-2.32	suppressor			
MBL2	-2.34	suppressor			
GSN	-2.42	suppressor			
TPM1	-2.46	suppressor			
PSRC1	-2.78	suppressor			
GPX3	-2.8	suppressor			
PERP	-3.42	suppressor			
MSX1	-3.5	suppressor			
MBD1	-4.15	suppressor			
BTG2	-5.26	suppressor			
KLF6	-5.67	suppressor			
EGR2	-6.03	suppressor			
EGR1	-15.88	suppressor			
					
24 month male mice (KO *vs*. WT)			24 month female mice (KO *vs*. WT)		

Name	Fold Change	function	Name	Fold Change	function

JUN	2.29	oncogene	LIMK1	4.9	oncogene
PTTG1	2.25	oncogene	ROS1	4.51	oncogene
PLK3	3.91	suppressor	CCND1	4.44	oncogene
BLNK	2.13	suppressor	APC	2.87	oncogene
GPX3	2.11	suppressor	MAFB	2.57	oncogene
BEXL1	2.08	suppressor	MYBL2	2.54	oncogene
WWOX	-2.00	suppressor	SH2B2	2.25	oncogene
PEG3	-2.2	suppressor	PDGFB	2.23	oncogene
WISP1	-3.48	suppressor	E2F3	2.08	oncogene
PERP	-4.03	suppressor	FLT4	2.02	oncogene
			MYCL1	-2.08	oncogene
			GPC1	-2.09	oncogene
			CDK5	-2.11	oncogene
			DCT	-2.3	oncogene
			SH3RF1	-2.35	oncogene
			NKX2-5	-2.42	oncogene
			BANP	3.97	suppressor
			PEG3	2.91	suppressor
			IFI16	2.63	suppressor
			BLNK	-2.55	suppressor
			DKK3	-4.13	suppressor
			GPR65	-6.82	suppressor

More genes altered expression patterns in male mutant mice compared with female mutant mice at 6 month of age (both in numbers and fold). For example, Jun, Fos, and Myc mRNA levels decreased by 2.87-, 5.74-, and 7.76-fold, respectively, in male mutant mice; on the other hand, tumor suppressor genes such as KLF6 (Kruppel-like factor 6), EGR2, and EGR1 (early growth response 2 and 1) were down-regulated by 5.67-, 6.03-, and 15.88-fold, respectively. KLF6 inhibits cell proliferation through up-regulation of p21 expression in liver cells [26]. EGR1 and 2, early transcription factors, increase apoptosis in tumor cells. The down-regulation of oncogenes indicated that in RXRα-deficient hepatocytes, the cell growth activity was compromised, providing another reason for the observed shortened cell lifespan due to RXRα deficiency. The same trend observed in tumor suppressor genes suggested that negative regulation of cell cycle was also impaired. For example, p21 is up-regulated by activation of RXR/RAR dimer in HepG2 cells [27]. Consistently, our data showed the decrease of p21 mRNA levels by 2.32 folds in 6 month old male mutant mice. BTG2 (B-cell translocation gene 2), a downstream effector of the p53 pathway [28], also had a 5.26 fold reduction. Several genes belonging to the p53 pathway had altered mRNA levels and lead to compromised p53 pathway activity. The impairment of the p53 pathway and other negative regulators implied that due to RXRα deficiency hepatocytes would surpass the cell cycle barrier more easily and be transformed into malignant cells. In female mutant mice, the mRNA levels of the above mentioned genes were not changed. One obvious trend the data points to is that all negative regulator genes for cell cycle transition decreased their mRNA levels in female mutant mice. It also implies cell cycle checkpoint machinery is impaired in female mutant mice.

Contrary to 6 month old female mutant mice, 24 month old female mutant mice had more genes with modified mRNA levels than did the male mutant mice of the same age group (22 vs. 10). In addition, no obvious trends in gene expression patterns were noted. Oncogenes and tumor suppressor genes were up or down regulated randomly.

The impact of RXR-mediated pathways on cell growth is very complex. It can be tissue- or cell type-specific. The activation of RXR/CAR, RXR/PXR, and RXR/PPARα pathways could induce hepatomegaly [29-31]. On the contrary, RXR/RAR or RXR/VDR pathways inhibit tumor cell growth [1,32]. It is likely that the complexity of changes seen in gene expression profiles reflect the net results of combined proliferative and anti-proliferative effects due to hepatocyte RXRα deficiency.

Our data implied that in matured livers, RXRα deficiency has more impact on cell growth-related gene expression levels in males than in females; but in aged liver, female mice have more changes in cell growth-related gene expression patterns than do male mice.

### Immune response-related genes

Immune response-related genes also had significant changes in mRNA level due to RXRα deficiency (table 7). At 6 month of age, male and female mutant mice had 15 and 4 genes with modified mRNA levels, respectively; however, at 24 month of age, there were 10 and 24 genes with change expression levels in male and female mice, respectively. In aged mice, the number of genes with altered mRNA levels increased significantly in female mutant mice. Another striking change in aged mice was that most of the immune response-related genes (8 out of 10 in males and 18 out of 24 in females) increased in mRNA levels. Such expression trends were not found in 6 month old mice, implying increased inflammation status might occur in both genders at old age due to RXRα deficiency.

**Table 7:** Fold changes of the mRNA levels of the immune response genes in male and female hepatocyte RXRα-deficient mouse livers.

6 month male mice (KO *vs*. WT)			6 month female mice (KO *vs*. WT)		
Name	Fold Change	function	Name	Fold Change	function

DSCR1	-5.50	immune response	CD9	-2.2	immune response
CXCL10	-4.47	immune response	CD24	-4.2	immune response
IKBKB	-3.31	immune response	HLA-G	-2.38	immune response
LIFR	-3.25	immune response	FSTL1	-2.14	immune response
IGHG1	-2.53	immune response			
			
S100A9	-2.39	immune response	24 month female mice (KO *vs*. WT)		
			
IGHM	-2.17	immune response	Name	Fold Change	function
			
MME	-2.17	immune response	B2M	-2.47	immune response
FCGR2B	-2.14	immune response	IL7	-2.38	immune response
CXXC5	2.01	immune response	IL17RD	-2.28	immune response
CCL19	2.04	immune response	LIFR	-2.07	immune response
TAX1BP1	2.13	immune response	HLA-G	-2.03	immune response
SAA4	2.18	immune response	NFATC2	-2.03	immune response
CXCL2	3.39	immune response	CD14	2.02	immune response
HLA-G	3.76	immune response	CD48	2.02	immune response
			FCER1G	2.05	immune response
			HLA-E	2.05	immune response
			NFATC4	2.06	immune response
			IL1RN	2.14	immune response
			
24 month male mice (KO *vs*. WT)			IGHM	2.23	immune response
			
Name	Fold Change	function	CD24	2.38	immune response
			
IL13RA1	-2.57	immune response	S100A9	2.4	immune response
HLA-G	-2.33	immune response	NFE2	2.42	immune response
DSCR1	2.01	immune response	IL12RB1	2.46	immune response
CCL7	2.03	immune response	LIF	2.6	immune response
NTRK3	2.23	immune response	IL1A	2.76	immune response
NTRK2	2.29	immune response	IL16	2.83	immune response
CXCL3	2.47	immune response	SLPI	2.97	immune response
CLEC2D	4.41	immune response	CD55	3	immune response
CXCL10	5.92	immune response	MARCO	3.24	immune response
IGH-1A	8.93	immune response	MEF2C	5.03	immune response

The RXR/PPAR dimer attenuates the inflammation response in colon [32]. RXR and PPAR agonists decrease TNFα (tumor necrosis factor α) and IL-1β (interleukin 1β) mRNA levels. In liver tissue, the acute response to external stimulus was associated with a down-regulation of RXRα expression [33]. Lipopolysaccharide (LPS) induces a rapid, dose-dependent decrease in RXRα, β, and γ proteins in hamster liver [33]. Alcohol induced pro-inflammation gene expression (TNFα, IL6, and IL1β) is enhanced due to hepatocyte RXRα deficiency [34]. These observations implied an inverse correlation between inflammation and RXRα signaling. These findings indicate that RXRα deficiency increases inflammation response to stimulus, which might be due to deregulation of a panel of immune-related genes. Furthermore, the impact of RXRα deficiency on immune response genes was more obvious in aged than in young mice.

### The impact of hepatocyte RXRα-deficiency on the expression of gender-dependent genes

The expression of many hepatic genes are gender dependent [35]. However, the findings might vary depending upon the age and strain of mice studied. The susceptibility of night blindness and xerophthalmia, the most common symptoms of vitamin A deficiency, are also gender dependent; the incidence is higher in males than females [36]. Thus, we studied the impact of RXRα deficiency on the expression of hepatic cancer-related genes that have a gender-dependent expression pattern. Our data showed that the numbers of gender-dependent genes in 6 month old wild type and hepatocyte RXRα-deficient mice are 329 and 200, respectively. When the mice were aged, the number of gender-dependent genes in wild type mice dropped significantly (127), but not so much in mutant mice (167) suggesting aging narrowed the gender gap more in wild type mice than in the mutant mice (table 8). There were common gender-dependent genes in both wild type and hepatocyte RXRα-deficient mice. The names of the genes that showed the greatest fold changes and those genes had the greatest fold changes due to RXRα deficiency at 6-month old age are listed (tables 9 and 10). Also at 24-month old, those genes showed the greatest fold change and genes had the greatest fold changes due to hepatocyte RXRα deficiency are listed in Tables 11 and 12. Surprisingly, the gender-dependent hepatic gene expression was also age-dependent as there was no overlap between the two age groups. Our data indicate that RXRα deficiency affects gender-dependent hepatic gene expression and that this effect is age-dependent.

**Table 8:** Number of cancer-related genes that showed gender-dependent (male vs. female) expression pattern in 6- and 24-month old wild type and hepatocyte RXRα-deficient mice

	Wild Type specific (A)	RXRα deficiency specific (B)	Overlap (C)	Wild type total (A+C)	RXRα deficiency total (B+C)
6-month	240	111	89	329	200
24-month	76	116	51	127	167

**Table 9:** Ten gender-dependent genes, which have the greatest fold changes, in wild type and hepatocyte RXRα-deficient 6-month old mice.

Male *vs*. Female in WT		Male *vs*. Female in KO	
Genes in A	Folds	Genes in B	Folds

DNAJB1	26.62	IL1R1	31.10
AFP	22.52	SOCS3	10.63
ATF3	19.34	FMN2	8.57
SERPINE1	14.99	CXCL14	6.95
HSPA1A	10.99	CAV1	6.00

PROM1	-8.55	WEE1	-4.98
CLCA1	-9.09	ID4	-5.50
DSCR1L1	-9.17	PML	-6.25
CYP2C19	-13.83	GAS1	-8.93
PEG3	-16.00	PDZRN3	-10.94

**Table 10:** Top ten gender-dependent cancer-related genes that have the greatest fold change due to hepatocyte RXRα deficiency.

Male *vs*. Female (6 month old mice)			
Genes in C	Folds (WT)	Folds (KO)	Fold change due to RXRα deficiency

MYC	33.49	5.52	6.07
CDKN1A	27.04	5.03	5.38
CYP17A1	-32.26	-10.32	3.13
BTG2	10.53	3.48	3.03
HSPA1B	23.96	8.35	2.87

ABCB1	-8.77	-19.23	0.46
B4GALNT1	-2.73	-6.06	0.45
PTP4A3	-4.33	-10.67	0.41
CXCL2	2.89	8.40	0.34
HSPB1	6.40	19.48	0.33

**Table 11:** Ten gender-dependent genes, which have the greatest fold changes, in wild type and hepatocyte RXRα-deficient 24-month old mice.

Male *vs*. Female in WT		Male *vs*. Female in KO	
Genes in A	Folds	Genes in B	Folds

SNCA	6.22	CLEC2D	45.07
SOX9	4.86	ASNS	27.09
CYP26A1	4.70	RB1CC1	11.82
ABCG2	4.01	FMN2	9.39
BCL6	3.46	RAMP2	4.13

PRLR	-3.98	ITGA6	-5.78
CXCL10	-5.29	DCT	-6.10
CD79B	-7.09	PSCDBP	-6.33
IL7	-9.71	PLA2G7	-6.45
DSCR1L1	-10.94	CD55	-13.57

**Table 12:** Top ten gender-dependent cancer-related genes that have the greatest fold change due to hepatocyte RXRα deficiency.

Male *vs*. Female			
Genes in C	Folds (WT)	Folds (KO)	Fold change due to RXRα deficiency

SERPINB1	-9.62	-2.41	3.99
CYP3A4	-43.86	-11.68	3.75
PROM1	-6.14	-3.13	1.96
CD74	-3.86	-2.02	1.91
OSGIN1	4.45	2.34	1.91

CAV1	2.14	3.74	0.57
IGHM	-2.28	-5.68	0.40
S100A8	-6.80	-21.10	0.32
MT1E	-2.03	-6.62	0.31
S100A9	-6.10	-31.35	0.19

## Conclusion

Collectively, RXRα deficiency could lead to significant changes in expression levels of cancer-associated genes in a gender- and age-dependent manner. Overall, there is increased resistance for apoptosis; increased cell migration activity; impaired cell protection ability; compromised cell cycle checkpoint machinery, and elevated inflammatory status. These changes may reflect the deregulation of multiple pathways in liver owing to RXRα deficiency. Although 24 month old hepatocyte RXRα-deficient mice do not develop spontaneous liver cancer, our data implied that hepatocyte RXRα-deficient mice might be more susceptible to cancer development, and this increased risk might be gender- and age-dependent manner.

The current study provides a database of cancer-related hepatic genes that might contribute to a difference in liver cancer incidence between genders as well as due to aging or retinoid signaling deregulation. The limitation of this study is that the role of those genes associated with liver cancer remains to be characterized in actual liver cancer models, which will be done in our future study.

## Methods

### Animals

Animal protocols were approved by the institutional animal use committee of the University of Kansas Medical Center, Kansas City. Male and Female C57BL/6J wild type and RXRα knock out mice were weaned at 28 days, housed individually, given free access to water, and randomly assigned to study groups. Four groups of mice were used to determine the effects on gene expression profile at two ages in both male and female mice. Each group had 3 mice. At particular time points after birth, 6 month (mature) and 24 month (aged) mice were sacrificed by cervical dislocation, and the livers were rapidly excised and flash frozen in liquid nitrogen. No signs of pathology were detected in any of the animals used.

### RNA Isolation and Preparation for Microarray Hybridization

Total RNA was isolated from frozen mouse livers using Trizol Reagent (Invitrogen Corporation, Carlsbad, CA) and further purified using the RNeasy Mini Kit (Qiagen Inc., Valencia, CA). Total RNA was quantified by UV spectrophotometry and its integrity and quality were assessed on RNA 6000 Nano LabChips with the Bioanalyzer 2100 (Agilent Technologies, Palo Alto, CA). Total RNA was reverse transcribed into cDNA using reverse transcription kit provided by Invitrogen Company. Synthesis and purification of double-stranded cDNA were conducted as described in the Expression Analysis Technical Manual (Affymetrix, Santa Clara, CA). Biotin-labeled cRNA was synthesized from the purified cDNA using the BioArray High Yield Transcript Labeling Kit according to the manufacturer's specifications (ENZO Life Sciences, Farmingdale, NY). Labeled cRNA was purified using the GeneChip Sample Cleanup Module, quantified by UV spectrophotometry and assessed for quality with the Bioanalyzer 2100. Twenty μg purified cRNA was fragmented and 15 μg fragmented cRNA was hybridized to Affymetrix Mouse Genome 430A 2.0 Array GeneChips (Affymetrix, Santa Clara, CA) according to the Expression Analysis Technical Manual. Washing and staining of the hybridized arrays were carried out using the Fluidics Station 400 and arrays were subsequently scanned with the Hewlett Packard GeneArray Scanner.

### Microarray Data Analysis

Affymetrix scan data (.cel files) were imported into Rosetta Resolver for analysis (Rosetta Biosoftware, Seattle, WA). Following intrachip normalization and background correction, individual replicates were combined into single "ratio experiments" by an error-weighted method using the control group as a baseline. An agglomerative hierarchical clustering algorithm utilizing an error-weighted Euclidian distance measure was performed on the ratio experiments to identify active genes. Transcripts were defined as active if they increased or decreased by greater than 2.0-fold. The microarray data from this work was submitted to the ArrayExpress database and the accession number is E-MEXP-1711.

### Confirmation of mRNA level changes by quantitative real-time PCR

The synthesized cDNA was diluted 20 fold by water. All primers and probes were designed based on nucleotide sequences in Genbank using the Primer Express software (PE Applied Biosystems). PCR reaction efficiency was calculated for each primer pair using with five dilution points of the calibrator sample to validate primers and probes. The PCR product covered at least two exons according to introns-exons organisation of selected genes. Each real-time PCR reaction consisted of 1× PCR Master Mix (PE Applied Biosystems), 0.5 μM forward and reverse primers and 1 uM corresponding probe. Final volume is 20 μl. Reactions were carried out on ABI PRISM 7900 real time PCR system (PE Applied Biosystems) for 40 cycles (95°C for 15 s, 60°C for 1 min). The expression fold change for each gene was calculated using the Ct method and β-actin was used as an internal control.

## Competing interests

The authors declare that they have no competing interests.

## Authors' contributions

Professor WYJ designed and supervised the project; HL bred mice and extracted hepatic RNA; GL and LML conducted the microarray experiment; GML performed the data analysis and wrote the manuscript. All authors read and approved the final manuscript.
